# Should you be worried?

**DOI:** 10.1007/s12471-014-0528-x

**Published:** 2014-02-13

**Authors:** J. Elias, A. R. Willems, A. A. M. Wilde

**Affiliations:** 1 Department of Cardiology, Sint Lucas Andreas Hospital, Jan Tooropstraat 164, 1061 AE Amsterdam, the Netherlands; 2 Academical Medical centre Amsterdam, Meibergdreef 9, Postbus 22660, 1100 DD Amsterdam, the Netherlands

**Fig. 1:**
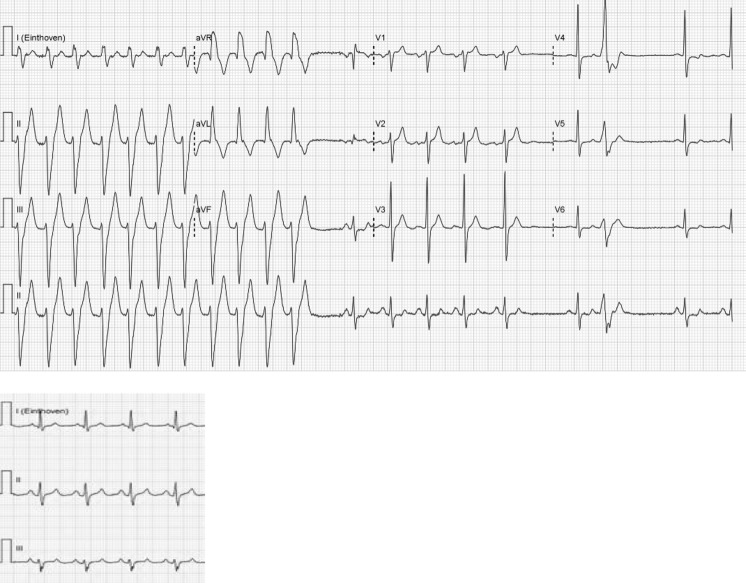
Twelve-lead ECG during and after conversion to sinus rhythm

The ECG in Fig. 1 shows a QRS tachycardia with a QRS duration of 146 ms and a frequency of 160 beats/min. There is a left axis deviation and a complete right bundle branch block. It is important to make a differentiation between ventricular tachycardia (VT) and supraventricular tachycardia (SVT). Signs suggestive of both a ventricular and supraventricular origin are present in our patient. The tachycardia was terminated by adenosine. This is (usually) suggestive of a supraventricular origin. So differential diagnostic paroxysmal SVT conducted with aberrancy is a possibility because of its relatively narrow QRS. The presence of ventriculoatrial dissociation in the electrocardiogram makes the diagnosis of VT stronger. If assuming a ventricular origin, VT originating in or near the Purkinje system (fascicular VT or idiopathic left ventricular VT) is likely.

In 1979, Zipes et al. were the first to identify the electrophysiological characteristics of an idiopathic VT. In 1981 Belhassen et al. described the use of verapamil in this VT, which is refractory to conventional treatment [1]. Therefore, this VT is commonly referred to as Belhassen, verapamil-sensitive or fascicular VT. It is characterised by a right bundle branch block pattern and left axis deviation (left posterior fascicle). A right axis deviation is less frequently noted.

The prevalence is unknown but patients are typically young and healthy when their first episode occurs. It is more common in males than females. The mechanism of the tachycardia is postulated to be triggered activity or reentry involving the left-sided Purkinje system and abnormal Purkinje or myocardial tissue [2].

The termination of fascicular VT usually requires intravenous antiarrhythmic medication. In patients with frequent or symptomatic fascicular VT long-term medication may be required. Patients with recurrent refractory episodes of VT may be referred for radiofrequency ablation. The prognosis of fascicular VT is good, in just a few cases it can result in a tachycardia-induced cardiomyopathy. Syncope and sudden death are extremely rare [3].
